# Toxicological and Biochemical Analyses of an Autopsy Case Involving Oral Overdose of Multiple Antidiabetic and Antihypertensive Drugs

**DOI:** 10.1155/2018/5864658

**Published:** 2018-11-25

**Authors:** Tomoya Ikeda, Naoto Tani, Shigeki Oritani, Alissa Shida, Yayoi Aoki, Fumiya Morioka, Takaki Ishikawa

**Affiliations:** ^1^Department of Legal Medicine, Osaka City University Medical School, Asahi-machi 1-4-3, Abeno, Osaka 545-8585, Japan; ^2^Forensic Autopsy Section, Medico-legal Consultation and Postmortem Investigation Support Center, C/o Department of Legal Medicine, Osaka City University Medical School, Asahi-machi 1-4-3, Abeno, Osaka 545-8585, Japan

## Abstract

Oral antidiabetics can cause fatal hypoglycemia; although they can be chemically identified and quantified, biochemical investigations are important for assessing the biological consequences of an overdose. Such cases of overdose involving oral antidiabetics may involve other drugs for treating lifestyle-related diseases, particularly antihypertensives. Here, we report a toxicological and biochemical investigation of drugs and biochemical profiles in a fatal overdose involving multiple oral antidiabetics and antihypertensives. A 55-year-old woman died about 2 days after the ingestion of around 110 tablets of antidiabetics and antihypertensives that had been prescribed for her husband. A forensic autopsy and histological analysis demonstrated no evident pathology as the cause of death. A toxicological analysis suggested hypoglycemia and an overdose of antihypertensives as well as the retention of antidiabetics and diuretics in the pericardial fluid. A relatively low pericardial amlodipine concentration was observed, which may have been the result of its long half-life (slower distribution and reduction rate) and/or possible affinity with the myocardium. In addition, a biochemical analysis indicated hypoglycemia, without increased serum insulin and C-peptide, but with increased glucagon levels, as the possible influence of glibenclamide overdose. These observations suggest the usefulness of a combination of toxicological and biochemical analyses in postmortem investigations involving a fatal overdose of such drugs.

## 1. Introduction

Approximately 20% of patients who use insulin or oral antidiabetic drugs present at the emergency room with hypoglycemia at least once during their life [1, 2]. Glibenclamide is a second-generation sulfonylurea that stimulates insulin secretion and is used to treat type 2 diabetes mellitus [3, 4]. Glucose-lowering medications that increase circulating insulin in a glucose-independent manner, such as insulin and sulfonylurea therapy, are the most common causes of hypoglycemia [5, 6]. Hypoglycemia has been reported in a metformin overdose and a homicidal poisoning with glibenclamide [7, 8].

Oral antidiabetics can cause fatal hypoglycemia; although they can be chemically identified and quantified, biochemical investigations are important for assessing the biological consequences of an overdose. Such cases involving an overdose of oral antidiabetics may involve ingestion of other lifestyle-related drugs, particularly antihypertensives. There are many reports of calcium channel blocker toxicity in clinical cases [9–16]. However, it is easy to overlook hypoglycemia in patients with type 1 diabetes who fall into a comatose state with no “alarm reaction” produced by adrenaline in response to hypoglycemia [17]. The present study reports a toxicological and biochemical investigation of the drugs and biochemical profiles in a fatality following the oral overdose of multiple antidiabetics and antihypertensives.

## 2. Case Report

A 55-year-old woman with a medical history of cystitis and anaphylactic shock ingested about 110 tablets of antidiabetics and antihypertensives (Lipitor®: atorvastatin calcium hydrate; Lasix® [furosemide] 20 mg; Diart® [azosemide] 60 mg; amlodipine 5 mg; Inhirokku® [cilazapril hydrate] 1 mg; and glibenclamide 2.5 mg; and Bratogen, among others.) that had been prescribed for her husband at 15:00 on November 18. At 05:00 on November 19, she had complaints of chest pain and was taken by ambulance to the hospital. She conveyed to the ambulance crew that she had consumed a large quantity of medications. The ambulance crew confirmed finding a number of empty drugs packets, including those for 10 tablets of Lipitor®, 10 tablets of Lasix®, 16 tablets of Diart®, 17 tablets of amlodipine, 27 tablets of Inhirokku®, and 21 tablets of glibenclamide. In the hospital, her blood glucose level was 36 mg/dL and her blood pressure was 80/38 mmHg. The doctor carried out an endoscopy, but found her stomach was empty. Therefore, gastric irrigation was not performed. The doctor prescribed Takepron® (lansoprazole) and Malfa® gel (aluminum hydroxide and magnesium hydroxide). After treatment, her consciousness became clear. She refused hospitalization and returned home. However, at 06:00 on November 20, about 40 h after consuming the medications, she went into cardiopulmonary arrest in her home. She was transported to the hospital by ambulance, and could not be resuscitated. A forensic autopsy was carried out to determine the cause of death about 18 h after death.

## 3. Autopsy Findings

**Figure 1:**
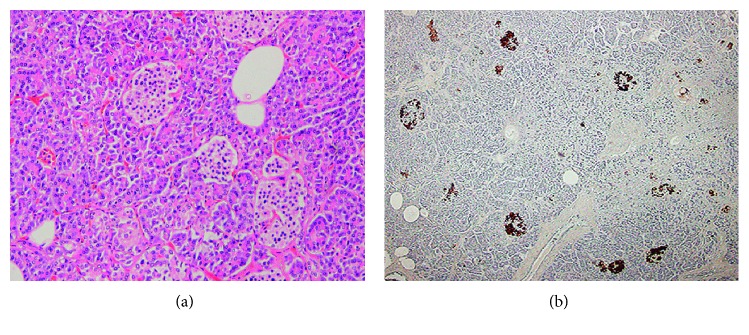
Microscopic findings. The pancreatic exocrine level ((a): hematoxylin-eosin staining, magnification ×100) was normal and antihuman insulin immunostaining of the pancreatic islets showed normal staining ((b): magnification ×40).

The postmortem progress during the autopsy was approximately 16–20 h. Her body height was 166 cm, body weight 51.7 kg, and body mass index 18.7 kg/m^2^. Macroscopic findings reported a heart weight of 290 g, frequent point bleeding on the posterior side of the heart wall, and edema. The lung weight was 350 g (right) and 340 g (left) with edema, and bleeding was observed within the pulmonary substance. The pancreas weighed 75 g and was edematous, except for the bleeding. All other organs were edematous; the weights of the liver and brain were 1375 and 1355 g, respectively. Microscopic findings showed moderate alveolus injury and alveolus hemorrhage in the lungs, suggesting intoxication. The pancreatic exocrine level was normal for histopathological changes, except for congestion and edema, and insulin immunostaining of the pancreatic islets was normal (Figure 1(a) and 1(b)). No other viscera showed a specific macro- or micropathology, except for the edema.

## 4. Toxicological Examination

### 4.1. Autopsy Materials

The left and right heart chambers, peripheral blood, pericardial fluid (PCF), bone marrow aspirate (BMA), and urine and stomach contents were routinely collected during autopsy using an aseptic syringe and analyzed in parallel. Approximately 12 mL peripheral blood was drawn from the external iliac vein. Approximately 14 mL PCF was drawn after opening the pericardial cavity. Approximately 5 mL BMA was collected by puncturing the lower thoracic vertebrae using a 10 mL syringe connected to a bone marrow needle (Jamshidi 11G; Baxter Healthcare Corp., McGaw, IL). Approximately 17 mL and 5 mL of urine and stomach contents were collected from the urinary bladder and gastric lumen, respectively. These specimens were subsequently stored at –20°C until analysis.

### 4.2. Gas Chromatography/Mass Spectrometry (GC/MS)

#### 4.2.1. Sample Preparation

Drugs were extracted using a Gilson ASPEC XL-274 automated solid/liquid phase extraction (SPE) instrument (Middleton, WI) as follows. To 0.5 mL of each sample, 50 *μ*L of a solution containing cocaine as the internal standard was added. Although cocaine is used extremely rarely in Japan, we first performed a drug screening examination to confirm that no cocaine was present. Next, we performed a fixed-quantity examination. We also investigated other drugs and chemicals by mixing standard drugs and internal standards with reference to previous research [18–20]. The pH of the sample was adjusted to approximately 7.0 by adding 6 mL of 0.1 M potassium phosphate buffer (pH 6.0). The mixture was then mildly stirred, centrifuged at 2,500 ×g for 5 min, poured into HF-Bond Elut Certify columns (Agilent Technologies, Tokyo, Japan), and gently aspirated. The columns had been previously conditioned with 2 mL methanol and 2 mL of 0.1 M potassium phosphate buffer (pH 7.0). After applying the samples, the columns were successively washed with 1 mL buffer, 1 mL of a 1 M acetic acid solution, and 1 mL methanol. Finally, the analytes were eluted with 3 mL of a freshly prepared mixture of chloroform/isopropanol (80 : 20) with 2% ammonium hydroxide. The eluates were collected and evaporated to dryness under a gentle stream of nitrogen at room temperature in a water bath. Residues were reconstituted in 50 *μ*L of ethylacetate, and 1 *μ*L aliquots of the extracts were analyzed.

#### 4.2.2. GC/MS Procedures

GC/MS was performed using an Agilent model 5975c MSD equipped with a DB-5MS column (length 30 m; id 0.25 mm; film thickness 0.25 *μ*m) (Agilent Technologies). The analysis conditions were as follows: column temperature, 100°C–325°C; injector temperature, 280°C; turbocharged carrier gas He at a flow rate of 48 cm/s; and an interface temperature of 300°C. Recovery of the standards range >95% at our facility. We added a known amount of standard to the matrix and applied the sample preparation and analytical methods to samples. Next, we calculated recovered amount based on the peak intensity obtained by injecting solvent solutions of the standard directly into the analytical instrument.

### 4.3. Liquid Chromatography/Mass Spectrometry/Mass Spectrometry (LC/MS/MS)

#### 4.3.1. Sample Preparation

To a 1.5 mL test tube, 25 *μ*L sample was added along with 1.25 *μ*L of the internal standard solution (1 *μ*g/mL final concentration) and 6.25 *μ*L of the extraction solution (acetonitrile:10 mM ammonium formate [pH 3.86] = 90 : 10). Cocaine was used as the internal standard. To the 50 *μ*L supernatant, 25 *μ*L of 10 mM KH_2_PO_4_ was added, and the sample solution was vortex-mixed for 10 s and then centrifuged at 15,000 rpm (7,500 ×g) for 3 min. The supernatant (70 *μ*L) was transferred to a high-performance liquid chromatography vial, and 20 *μ*L of the clear supernatant was directly injected into the LC/MS/MS system for analysis. The total running time was 2 min for each injection.

#### 4.3.2. LC/GC/MS Procedures

Automated LC/MS/MS following SPE was performed using a Shimadzu Prominence ultrafast liquid chromatography system (Kyoto, Japan; column, 2.1 m × 150 mm i.d., L-column2 ODS; sample; column temperature, 40.0°C; flow rate, 0.2 mL/min; injection vol.: 20 *μ*L; mobile phase, A: 10 mM ammonium formate + 5% methanol and B: 10 mM ammonium formate + 95% methanol), and an AB Sciex mass spectrometer (4000QTRAP; interface: TurboV source with electrospray ionization (ESI); Takara Bio, Inc., Shiga, Japan). A triple quadrupole mass spectrometer equipped with an ESI source was used for mass analysis and detection. The acquisition mode was performed in multiple reaction monitoring (ESI+; amlodipine/glibenclamide, IonSpray [IS]: voltage: 5500 V, RT = 0–10 min, ESI–; furosemide, IS voltage 4500V, RT = 10 min). Q1/Q3: amlodipine; 409.0/238.1 glibenclamide; 494.2/396.2 furosemide; 329.0/204.9 [21, 22].

### 4.4. Validation Information

We performed both general screening and direct searching. One quantifier (target) ion and more than two qualifier ions were selected for each drug. A calibration curve was obtained by plotting the peak area ratio of the drug to the internal standard versus the amount of drug using MSD ChemStation (Agilent Technologies). The limit of detection (LOD) was estimated with a signal-to-noise (S/N) ratio of 3 (S/N = 3) for transition with the lowest intensity. The limit of quantification (LOQ) was the lowest concentration of analyte that could be specifically measured in three transitions. The LOD and LOQ for cocaine were 50 ng/mL and 200 ng/mL, respectively. The retention time and mass spectrum of each drug were obtained at the level of 1 μg/mL. Data were obtained for each drug, including retention time, qualifier ion/target ion percentage, mass spectrum, and calibration curve. When a drug level detected by this method was very high, we diluted the sample and performed requantification. As a result, the calibration carve was not weighted. Although the matrix effects were unclear in the postmortem test results, the reproducibility was high (>95%), which suggested that the detected values had high reliability. Our analyses were based on our previously reported methods [21–25].

## 5. Results

### 5.1. Biochemical Investigation

A postmortem biochemical investigation was performed immediately after the autopsy. No preservatives such as sodium fluoride were used in the investigation. The results showed low glucose levels (1–5 mg/dL) in the blood and body fluids without increased insulin (1.34–10.4 *μ*g/mL) or C-peptide (0.25–1.41 ng/mL), accompanied by slight-to-moderately decreased glucagon levels (Table 1). Glucose and lactate levels were 1 mg/dL and 1 mmol/L in vitreous humor and cerebrospinal fluid (CSF), respectively (Table 1).

The serum chloride concentration was low (71–81 mEq/L), but otherwise, no electrolyte disorders were significant. Serum renin and aldosterone were not elevated, but angiotensin I and II were, showing dissociation. The brain natriuretic peptide (BNP) level in PCF remained low, but the atrial natriuretic peptide (ANP) level was slightly increased (Table 2). Tables 1 and 2 show the autopsy reference values [26].

### 5.2. Toxicological Analysis

**Figure 2:**
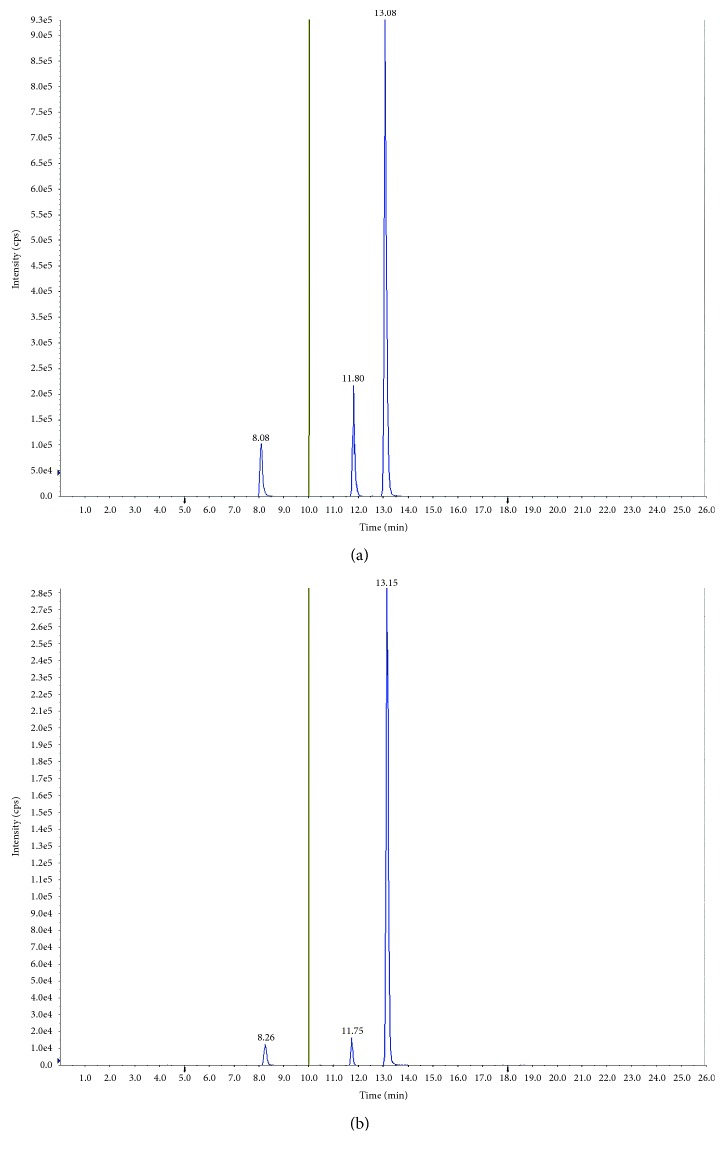
Multiple reaction monitoring chromatogram of a standard sample (1–100 *µ*g/mL) adjusted to concentration (a) and pericardial fluid (b). Retention times: furosemide: 8.2 min; amlodipine: 11.8 min; glibenclamide: 13.1 min, 10 min negative mode, and 10 min positive mode.

The toxicological analysis using GC/MS and LC/MS/MS detected lidocaine (4.97 *μ*g/mL in heart blood and 0.12 μg/mL in urine), which was used when the patient was receiving intensive care at the hospital, as well as glibenclamide, amlodipine, and furosemide at 81, 190, and 19 ng/mL in right heart blood; 54, 72, and 16 ng/mL in peripheral blood; and 280, 55, and 100 ng/mL in PCF, respectively. Glibenclamide and amlodipine were also detected in BMA (50 ng/mL and 2.5 ng/mL), urine (2 ng/mL and 320 ng/mL), and stomach contents (3 ng/mL and 240 ng/mL), respectively; however, furosemide levels could not be determined owing to the limited quantities of these specimens (Table 3, Figure 2). The autopsy reference levels are shown in Table 3 [27, 28]. Although we performed a screening examination using GC/MS, we were only able to assay lidocaine. Other drugs were detected using LC/MS/MS.

## 6. Discussion

To the best of our knowledge, no such cases of a suicide involving a megadose of glibenclamide have been reported. Therefore, we conducted a comparison with general fatal concentrations. In the present case, the glibenclamide level was 280 ng/mL in the PCF, although the therapeutic or maximum concentration in a previous survival case was 191 ng/mL [27, 28]. Therefore, based on the measurement results from the present case, we considered that amlodipine, glibenclamide, and furosemide had a slight-to-severe adverse effect on circulatory insufficiency.

However, a case involving a 42-year-old female who survived the acute ingestion of 50–100 mg of amlodipine and manifested hypotension, tachycardia, and pulmonary edema has been reported. Her initial plasma amlodipine level of 88 ng/mL at 2.5 h postingestion gradually declined to 79 ng/mL after another 35 h [29]. Three other adults have survived the ingestion of 100–1000 mg of amlodipine after attaining peak plasma levels of 67–393 *μ*g/L [9, 30, 31].

Therefore, in terms of amlodipine only, we considered the possibility that the present case did not reach fatal levels in iliac venous blood, leading us to conclude, based on the severe hypoglycemia caused by oral glibenclamide, similar to noninsulin therapeutic drugs for diabetes, that the cause of death in the present case was subacute metabolism disorder. Other drugs such as amlodipine could have hastened death in the present case. Glibenclamide is hepatically metabolized to active metabolites, which are renally excreted. These metabolites may accumulate in patients with renal dysfunction, thereby increasing the risk of hypoglycemia, which is defined as a blood glucose level <70 mg/dL [32, 33]. In clinical case reports, glibenclamide was associated with a 52% greater risk of experiencing at least one episode of hypoglycemia compared with other secretagogues and an 83% greater risk compared with other sulfonylureas [34, 35]. Hypoglycemia can be classified into two groups: one that arises from the action of the autonomic nervous system and one that is related to an insufficient supply of glucose to the brain (neuroglycopenia) [36]. In the present case, the biochemical data indicated hypoglycemia, without increased serum insulin and C-peptide, but with increased glucagon levels, as the possible influence of glibenclamide overdose. In addition, we found that glucose and lactate levels had decreased in the CSF and vitreous humor. However, to our knowledge, no scientific evidence regarding the stability of glucose in the CSF and vitreous humor has been reported [37, 38].

In the present case, decreased glucagon levels were observed in the blood and PCF [39]. On the other hand, the evaluation of postmortem blood sugar levels was difficult. In the present case, postmortem blood sugar levels were uneven. However, blood sugar levels may become higher than 500 mg/dL in the case of acute stress (acute death), and hypoglycemia (20 mg/mL or less) has been found in septic shock cases [40].

Amlodipine is a dihydropyridine calcium channel blocking agent. It has pharmacokinetic properties: a half-life of about 30 h and oral bioavailability approaching 100% [41]. After oral administration and absorption, the drug is detectable within 0.5–1 h, but the peak concentration is not reached until a mean time of approximately 8 h [41].

The toxicological data suggested an overdose of glibenclamide and amlodipine, as well as the retention of glibenclamide and furosemide in the PCF. The half-life of glibenclamide is approximately 2.7 h [42]. On the other hand, the metabolism of the PCF is necessary to maintain appropriate osmotic pressure [43]. In the present case, glibenclamide concentrations were low, except in the PCF. Therefore, we suspected that glibenclamide was metabolized faster during the excretion period. A drug's absorption or excretion period can be estimated by measuring its levels in all body fluids. Therefore, drug levels should be measured in all body fluids. In other words, the relatively low pericardial amlodipine concentration may have been the result of its long half-life (slower distribution and reduction rate) and/or a possible affinity to the myocardium. However, there were no remarkable macro- or micropathological findings in the heart in the present case.

In addition, the biochemical data indicated hypoglycemia as a possible influence of glibenclamide overdose intoxication, without increased serum insulin and C-peptide, but with increased glucagon levels. C-peptide is more stable than insulin, the concentration of which slightly decreases after death. In addition, the half-lives of insulin and C-peptide are short, which means that in this case, there may have been no detectable increases at 40 hours after internal administration [44]. Dissociation of angiotensin and renin or aldosterone may have been a consequence of the short-loop negative feedback mechanism under the prolonged influence of amlodipine overdose. The low BNP and slightly increased ANP levels in the PCF suggested prolonged hypotension with elevated atrial pressure [24]. This finding suggests that amlodipine did not cause a continuous or severe heart burden. Moreover, the effect of furosemide overdose on electrolytes was not identified. These observations suggest the usefulness of a combination of toxicological and biochemical analyses in postmortem investigations involving an overdose of these drugs.

On the other hand, we cannot easily explain the pharmacologic reactions resulting from taking various kinds of drugs with different efficacies at the same time; therefore, further examinations are needed.

## Figures and Tables

**Table 1:** Biochemical analysis and reference data of the pericardial fluid, heart peripheral iliac vein blood, cerebrospinal fluid, and vitreous humor.

		HbA1c mmol/mol	Glucose (mg/dL)	Lactate (mmol/L)	Insulin g/mL	C-peptide (ng/mL)	Glucagon pg/mL
Present case	Left heart blood		1		217	0.69	214
	Right heart blood	33	5		1.34	0.25	
	Iliac vein blood		2		3.30	1.41	
	Pericardial fluid		4		2.09	0.07	109
	Vitreous humor (left/right)		1/1	136/94	3.0/3.0	0.3/0.3	50/50
	Cerebrospinal fluid		1	236	10.4	0.40	50

Autopsy reference values and medians (25th and 75th percentiles) in our department	Left heart blood	—	70 (18–186)	—	—	—	—
	Right heart blood	42 (41-42)	189 (72–361)	—	3.19(1.12–4.61)	—	353(208.7–692.5)
	Iliac vein blood	—	63 (26–141)	—	3.3(0.3–5.12)	—	—
	Pericardial fluid	—	97 (51–178)	—	—	—	—
	Vitreous humor	—	16.32	165.82	—	—	—
	Cerebrospinal fluid	—	—	—	—	—	—

**Table 2:** Biochemical analysis of the pericardial fluid, heart, and peripheral iliac vein blood, cerebrospinal fluid, and vitreous humor.

Present case	Chloride (CI) (mEq/L)	Potassium (K) (mEq/L)	Sodium (Na) (mEq/L)	Calcium (Ca) (mEq/L)	Renin (pg/mL)	Aldosterone (pg/mL)	Angiotensin I (pg/mL)	Angiotensin II (pg/mL)	ANP (pg/mL)	BNP (pg/mL)
Left heart blood	81	19.8	135	10.9	3	163	480	90	—	—
Right heart blood	71	33.9	125	10	6		500	110	—	—
Iliac vein blood	71	36.2	125	10.4					—	—
Pericardial fluid	102	25.2	133	7.9		16.2			47.3	211
Vitreous humor (left/right)	170/280	12.0/18.0	180/180	2.0/0.0						
Cerebrospinal fluid	146	4.0	153	0.7						

*Autopsy reference values and medians (25th and 75th percentiles) in our department*										
Left heart blood	87 (80–94)	24.9 (18.8–32.3)	128 (118–136)	10.7 (9.7–12.0)	—	—	—	—	0 (0–10)	8.1 (2.1–18.5)
Right heart blood	80 (73–88)	32.6 (25.8–41.5)	121 (111–130)	10.4 (9–11.7)	—	—	—	—	0 (0–10)	15.7 (2–58.4)
Iliac vein blood	83 (75–90)	33 (25.9–41.4)	127 (117–136)	12.0 (10.3–13.0)	—	—	—	—	5.2 (0–10)	4.8 (2–24.1)
Pericardial fluid	97 (89–105)	29.2 (23.6–40)	124 (113–133)	8.6 (7.8–9.4)	—	—	—	—	14 (9–28)	78.9 (17.7–308.0)
Vitreous humor (left/right)	—	—	—	—	—	—	—	—	—	—
Cerebrospinal fluid	—	—	—	—	—	—	—	—	—	—

**Table 3:** Toxicological analysis of the heart blood, pericardial fluid, iliac vein blood, urine, bone marrow, and stomach contents.

Drug	Amlodipine	Glibenclamide	Furosemide	Lidocaine
Therapeutic or max concentration of survival cases	1–24 ng/mL	191 ng/mL	0.001–0.010 ng/mL	1.5–5.0 *μ*g/mL
Right heart blood	190 ng/mL	81 ng/mL	19 ng/mL	4.974 *μ*g/mL
Pericardial fluid	55 ng/mL	280 ng/mL	100 ng/mL	
Iliac venous blood	72 ng/mL	54 ng/mL	16 ng/mL	
Urine	320 ng/mL	2 ng/mL		0.121 *μ*g/mL
Stomach	240 ng/mL	3 ng/mL		
Bone marrow	2.2 ng/mL	50 ng/mL		

## Data Availability

All data used to support the findings of this study are included within the article.
